# In Silico, In Vitro, and Pharmacokinetic Studies of UBMC-4, a Potential Novel Compound for Treating against *Trypanosoma cruzi*

**DOI:** 10.3390/pathogens11060616

**Published:** 2022-05-24

**Authors:** Christian Bustamante, Andrés Felipe Díez-Mejía, Natalia Arbeláez, Maurilio José Soares, Sara M. Robledo, Rodrigo Ochoa, Rubén E. Varela-M., Marcel Marín-Villa

**Affiliations:** 1 PECET-Programa de Estudio y Control de Enfermedades Tropicales, School of Medicine, Universidad de Antioquia, Medellín 050010, Colombia; andresfelipediez@gmail.com (A.F.D.-M.); natyac182@gmail.com (N.A.); sara.robledo@udea.edu.co (S.M.R.); 2 Cell Biology Laboratory, Carlos Chagas Institute/Fiocruz, Curitiba 81350-010, Paraná, Brazil; mauriliojsoares@gmail.com; 3 Biophysics of Tropical Diseases, Max Planck Tandem Group, Universidad de Antioquia, Medellín 050010, Colombia; rodrigo.ochoa@udea.edu.co; 4 Grupo (QUIBIO), School of Basic Sciences, Universidad Santiago de Cali, Cali 760032, Colombia; rubenevm@hotmail.com

**Keywords:** Chagas disease, *Trypanosoma cruzi*, AKT-like serine/threonine kinase, drug discovery, molecular docking, UBMC-4 inhibitor, pharmacokinetics

## Abstract

The lack of therapeutic alternatives for the treatment of Chagas disease, a neglected disease, drives the discovery of new drugs with trypanocidal activity. Consequently, we conducted in vitro studies using UBMC-4, a potential *Trypanosoma cruzi* AKT-like pleckstrin homology (PH) domain inhibitory compound found using bioinformatics tools. The half effective concentration (EC_50_) on intracellular amastigotes was determined at 1.85 ± 1 μM showing low cytotoxicity (LC_50_) > 40 μM on human cell lines tested. In order to study the lethal effect caused by the compound on epimastigotes, morphological changes were assessed by scanning and transmission electron microscopy. Progressive alterations such as flagellum inactivation, cell size reduction, nuclear structure alteration, condensation of chromatin towards the nuclear periphery, vacuole formation, and mitochondrial swelling with kinetoplast integrity loss were evidenced. In addition, apoptosis-like markers in *T. cruzi* were assessed by flow cytometry, demonstrating that the effect of UBMC-4 on *T. cruzi* AKT-like kinase reduced the tolerance to nutritional stress-triggered, apoptosis-like events, including DNA fragmentation, mitochondrial damage, and loss of plasma membrane integrity. After this, UBMC-4 was formulated for oral administration and pharmacokinetics were analyzed in a mouse model. Finally, upon oral administration of 200 mg/kg in mice, we found that a UBMC-4 plasma concentration remaining in circulation beyond 24 h after administration is well described by the two-compartment model. We conclude that UBMC-4 has an effective trypanocidal activity in vitro at low concentrations and this effect is evident in *T. cruzi* cell structures. In mice, UBMC-4 was well absorbed and reached plasma concentrations higher than the EC_50_, showing features that would aid in developing a new drug to treat Chagas disease.

## 1. Introduction

American trypanosomiasis or Chagas disease is a potentially life-threatening illness caused by the parasite *Trypanosoma cruzi*, a protozoan of the Kinetoplastida order whose transmission to humans is primarily vector-borne by triatomine hematophagous insects belonging to Reduviidae family. However, infection may also occur upon consumption of food contaminated with the parasite, transplacentally, via breastfeeding, transfused blood or donated organs. In Latin American and the Caribbean region, Chagas disease is endemic in 21 countries, affecting approximately 6–8 million people. Worldwide, Chagas disease causes 50,000 deaths per year, and 8000 newborns become infected during gestation. It is estimated that approximately 70–120 million people in the Americas live in areas of exposure and are at risk of contracting this disease [1,2].

Currently, there is no effective vaccine available on the market for preventing Chagas disease, and there are only two drugs endorsed by the WHO for the treatment of trypanosomiasis: nifurtimox and benznidazole (bz), both described as nitro-heterocyclic compounds, which are considered prodrugs, as metabolism by enzymes of the nitro reductase type is required to develop their trypanocidal activity. These drugs have been used as first-line treatments for more than 50 years and both have been described as having activity against the trypomastigotes and amastigotes forms of *T. cruzi* in the early phase of the disease. Despite benznidazole having been characterized as a compound with limited effectiveness and the slow pace of progress in research related to the mechanisms of action of this compound, this is currently the most frequently used drug due to a better availability in America compared to that of nifurtimox [3]. Both compounds exhibit noticeable limitations, including long-term treatments, issues related to safety and tolerability, and little or insufficient efficacy to cure chronic Chagas disease [4]. Side effects from benznidazole and nifurtimox are estimated to cause 15–20% of patients to discontinue the treatment. On the other hand, it has been observed that both medications are better tolerated in children than in adults, making it clear that the frequency and severity of side effects tend to increase with the age of the patient [5,6,7,8]. These drugs may cause different side effects such as peripheral neuropathy, anorexia, insomnia, polyneuropathy, nausea or vomiting, and their use is discouraged during pregnancy and lactation [9].

In the past decade, clinical trials for Chagas disease have tested limited new drugs. In the ClinicalTrials.gov database, there are 75 clinical trials registered for Chagas disease. Some of these trials have tested different treatment schemes of benznidazole and nifurtimox. These new dosing regimens are designed to reduce drug exposure in order to minimize adverse effects while maintaining efficacy. Other drugs tested, with activity against the parasite and/or treat cardiomyopathy caused by the disease, are amiodarone, carvedilol, and valsartan. Lastly, some trials have tested azoles such as posaconazole, ravuconazole, and fexinidazole, but none of them has shown effectiveness more significant than that of benznidazole [10,11,12,13]. Considering the current landscape for the drugs available to treat Chagas disease and the imminent risk of their obsolescence due to the increasingly frequent events of drug resistance in parasite populations, the search for new therapeutic alternatives that mitigate the effects caused by this disease is necessary [14,15]. In this regard, one of the most relevant goals to design new drugs is selecting targets and mechanistic pathways other than those described for current drugs, thereby limiting resistance. Three strategies have been proposed for the development of new drugs used for the treatment of neglected diseases: the first strategy is based on the development of compounds that interfere with or modulate key, vital proteins or enzymes for parasites, such as peptidases [16,17], enzymes that catalyze purines and pyrimidines such as aspartate transcarbamoylase [18], trypanothione reductase (TR) and the iron-containing superoxide dismutase (Fe-SOD), which protect the parasite against oxidative damage by reactive oxygen species [19]. Additionally, human proteins that have low identity with their orthologous proteins in parasites are the subject of studies intended to carry out screening through docking and molecular dynamics of modulator compounds of the activity of such proteins [20,21,22]. A second strategy consists of using vegetable- or marine-sourced natural products or other organic substances starting from fractions that contain pools of compounds sharing a similar chemical nature, in particular chemical candidates called active ingredients that inhibit the growth of parasites in vitro. [23,24,25]. 

Finally, one of the strategies that can generate higher success rates is the use of second-use medications supported for the treatment of other diseases. Particularly, they are used as parasite growth inhibitors [26]. Some of the targets of interest in this field are those related to enzymatic functions such as anabolism or catabolism of molecules, receptor proteins responsible for signaling such as kinases, transcription factors, ion channels, and protein transporters, among other types of macromolecules as targets of DNA and RNA [27].

Making use of the approaches above, molecular and cellular studies of *Leishmania* spp. and *T. cruzi* AKT-like serine/threonine kinase performed in our laboratory have led to results supporting a potential role of this enzyme as a drug target in the treatment of leishmaniasis and Chagas disease [28,29,30,31]. AKT, also known as PKB, is a pivotal molecule that mediates the PI3K/AKT/mTOR signaling pathway, known to play a key role in signal transduction pathways activated in response to growth factors or insulin, thereby contributing to different cell functions such as nutrient metabolism, cell growth, and apoptosis in humans [32]. These kinases are attractive pharmacological targets due to their key role in tumor cell survival or proliferation and they are overexpressed or activated in many human cancers [33]. Specific inactivation of AKT triggers apoptotic processes without affecting normal cells, making them a significant chemotherapeutic target, and AKT kinases have become increasingly interesting in pharmaceutical research over the past several years [34]. The *T. cruzi*, AKT-like serine/threonine kinase (TcAKT) enzyme has three functional domains: a pleckstrin homology (PH) domain at the N-terminal region, a catalytic domain (AGC), and a regulatory domain (PK). The PH domain is important as it has been shown to play a role in the high-affinity, specific interaction with membrane lipids such as phosphatidylinositol (3,4,5), trisphosphate (PtdIns(3,4,5)P3), and phosphatidylinositol(3,4)bisphosphate (PtdIns(3,4)P2), thus favoring enzyme anchoring to cell membranes and enhancing their phosphorylation at residues required for activation and localized to the catalytic domain and C-terminal hydrophobic region [32]. In trypanosomatids, the PI3K/AKT/mTOR pathway has only been partially characterized in experiments, although some computational studies suggest the existence of more kinases associated with this pathway [30,35]. 

On the other hand, it is important to mention that despite the similarity between *Leishmania* spp. and *T. cruzi* AKT-like kinase with its ortholog enzyme in humans, its PH domain exhibits a notable difference in the amino acid sequence, which allowed us to search for specific inhibitors that do not affect the protein in humans. Taking advantage of this difference, we performed a preliminary molecular docking-based strategy for the virtual screening of a library from the ZINC database. This search allowed the identification of a small organic compound potentially capable of interacting with the PH domain and received the name UBMC-4 [28,29,31].

In this study, UBMC-4 trypanocidal activity is presented from a specific docking against the PH domain where the protein-ligand interaction capacity was tested. Furthermore, the in vitro trypanocidal effect on *T. cruzi* amastigotes was determined (EC_50_) and the morphological and ultrastructural changes caused by this compound on the parasite epimastigotes were analyzed. Apoptosis markers in nutritionally stressed UBMC-4-treated *T. cruzi* were assessed by flow cytometry, reporting apoptosis-like events that include DNA fragmentation, mitochondrial damage, and loss of membrane integrity. Moreover, to carry out future UBMC-4 in vivo efficacy assays for the treatment of Chagas disease, we developed a formulation that permits oral administration and assessment of the pharmacokinetic profile in a murine model. Pharmacokinetic analyses were performed to investigate if UBMC-4 is absorbed after oral administration as well as the plasma concentration and the estimation of the dose required to achieve desirable plasma concentrations [36]. 

## 2. Results

### 2.1. Molecular Docking of UBMC-4 and the T. cruzi AKT-like Pleckstrin Domain

**Figure 1:**
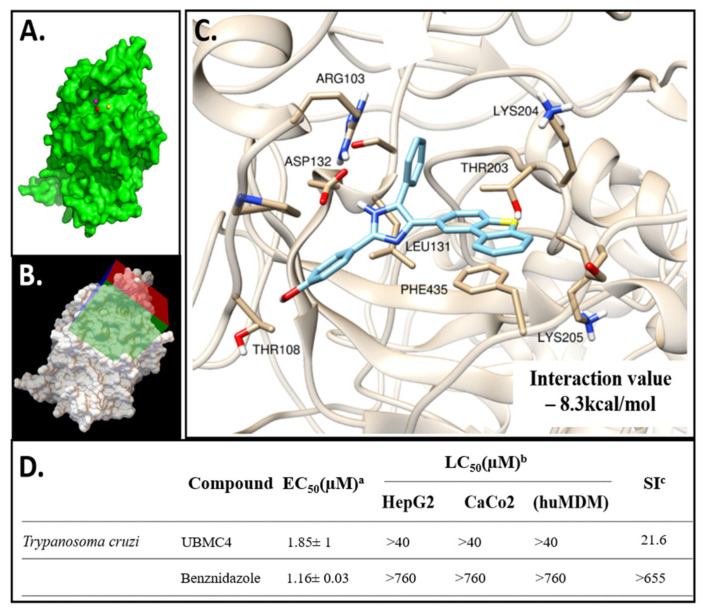
Structural modeling and molecular dynamics of the AKT-like protein in *T. cruzi*. (**A**) Structural model of the AKT-like protein in surface format. The pocket predicted by MetaPocket 2.0 server tools is presented with colored spheres. (**B**) Search box configured in the AutoDock Tools program for molecular docking of the predicted pocket near the protein PH domain with ~640,000 compounds in 3D format, executed on the DrugDiscovery@TACC server (drugdiscovery.tacc.utexas.edu). (**C**) UBMC-4 interacting pocket; key residues undergoing a certain molecular interaction with the compound and the interaction energy obtained are shown. (**D**) Cytotoxic and trypanocidal activity of the UBMC-4 compound compared with the reference drug benznidazole. ^a^ EC_50_; half maximal effective concentration for *T. cruzi* amastigotes. ^b^ LC_50_; median lethal concentration for macrophage-derived monocytes (huMDM); HepG2 liver tissue cell line and colon tissue cell line (CaCo2). ^c^ IS; selectivity index between (huMDM) and *T. cruzi* amastigotes. Data shown represent the average value of the experiments ± the standard deviation.

Once the docking model of UBMC-4 with the *T. cruzi* AKT-like protein was completed, an interaction with a set of key residues including Arg103, Leu131, and Lys204, (all three located near the enzyme PH domain) was found (Figure 1C). Classic hydrogen bonds and hydrophobic interactions between the PH domain and the ligand are the major contributors to a UBMC-4 score of −8.3 kcal/mol predicted by Vina. This model can become a strategy to improve the chemical structure of the inhibitor in a further stage of development for UBMC-4 if the mechanism proposed is validated through additional methods.

### 2.2. UBMC-4 Cytotoxic and Trypanocidal Activity

The UBMC-4 inhibitor’s trypanocidal activity was strong with an EC_50_ slightly higher than that of benznidazole, which was used as control (Figure 1D). UBMC-4 showed an EC_50_ for *T. cruzi* Tulahuen strain amastigotes of 1.85 ± 1 μM (0.774 µg/mL) while benznidazole showed an EC_50_ of 1.16 ± 0.03 μM for the same strain. In addition, UMBC-4 exhibited low cytotoxicity with a LC_50_ > 40 μM for monocyte-derived macrophages (huMDM), the liver cell line HepG2, and colon cell line CaCo2. The selectivity index (SI) between huMDM and *T. cruzi* amastigotes was 21.6. (Figure 1D). These data represent the average values and standard deviation for three experiments.

### 2.3. T. cruzi Is More Sensitive to Nutritional Stress in the Presence of AKT-like Inhibitor

**Figure 2:**
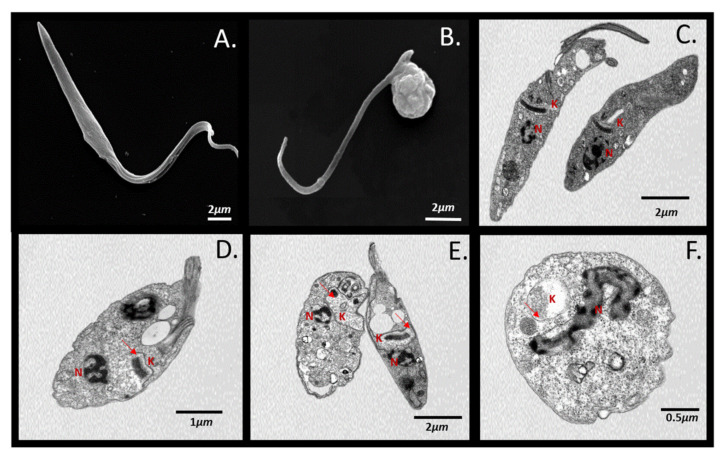
SEM and TEM of *T. cruzi* epimastigotes undergoing nutritional stress and inhibition of AKT-like protein. (**A**) SEM of epimastigotes undergoing nutritional stress. (**B**) SEM of epimastigotes undergoing nutritional stressand inhibition of AKT-like protein for 6 h. (**C**) TEM of epimastigotes undergoing nutritional stress. (**D**–**F**) TEM of epimastigotes undergoing nutritional stress and inhibition of AKT-like protein for 6 h. N, nucleus; K, kinetoplast; arrows indicate the place of mitochondrial alteration.

The impact of nutritional stress (culture medium substitution for PBS over 6 h) on the morphology and ultra-structure of *T. cruzi* epimastigotes exposed to concentrations of 10 µM UBMC-4 was evaluated. Parasite morphology and ultra-structure were not affected by nutritional stress itself. Specifically, apoptosis-like markers such as DNA damage and mitochondrial or cell membrane integrity were not found (Figure 2A,C). However, parasites undergoing nutritional stress in presence of UBMC-4 evidenced progressive alterations such as flagellum inactivation, cell size reduction, nuclear structure alteration, condensation of chromatin towards the nuclear periphery, vacuole formation, mitochondrial swelling with kinetoplast integrity loss, and other external and internal morphological changes including formation of possible blebs (Figure 2B,D–F).

**Figure 3:**
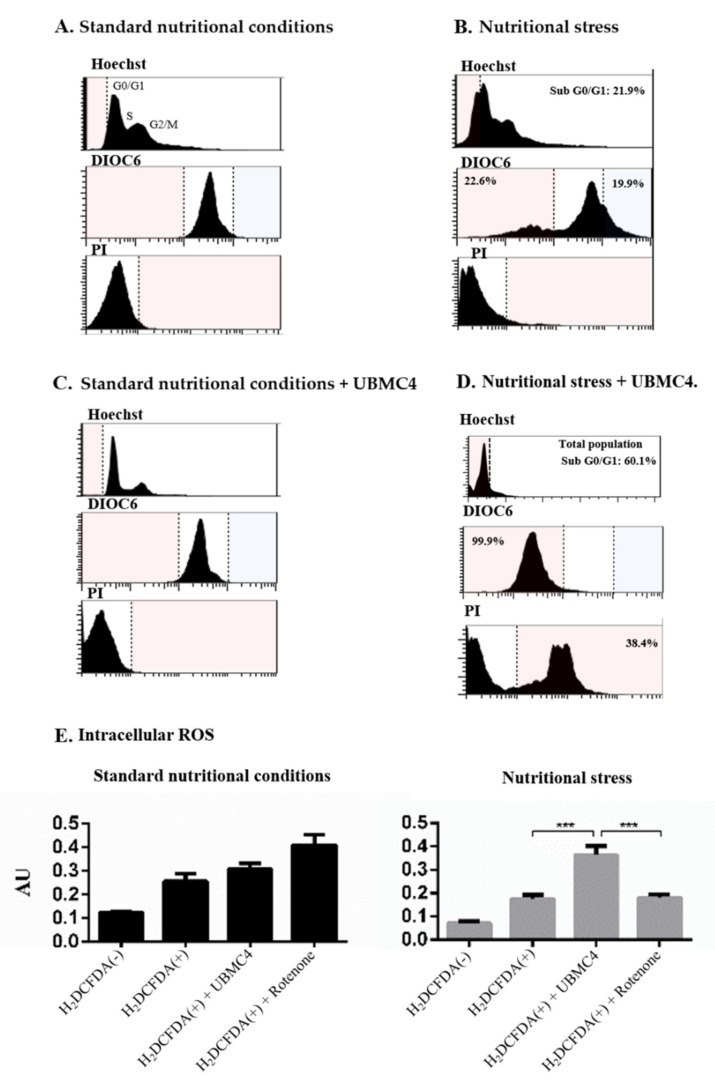
Determination of apoptosis-like markers in *T. cruzi* epimastigotes subjected to nutritional stress and inhibition of AKT-like protein. Frequency histograms for Hoechst (cell cycle), DiOC6 (Δ*Ψm*), and PI (cell membrane alteration) parameters. Flow cytometry analysis of epimastigotes grown for 24 h under standard (**A**) or nutritional stress (**B**) conditions inhibition of AKT-like protein under standard (**C**) or nutritional stress (**D**) conditions. Determination of intracellular ROS concentration in epimastigotes under standard and nutritional stress conditions with and without inhibition of the AKT-like protein for 24 h (**E**). Plots represent tendencies for three biological replicates per condition, and percentages are equivalent to the average obtained from three replicates. Asterisks indicate statistically significant differences with respect to the values for control treatments (*** *p* < 0.0004).

Nutritional stress caused parasite DNA fragmentation in 21.9% of the population (hypodiploid), promoted mitochondrial depolarization in 22.6%, and induced cell membrane damage in 2.27% of the parasite population compared with parasites cultured in normal nutritional conditions (Figure 3A,B). Accordingly, no changes in apoptosis-like markers were seen when using 10 µM UBMC-4 under normal culture conditions (Figure 3C). However, addition of UBMC-4 to parasite cultured under nutritional stress induced a progressive and gradual DNA fragmentation in up to 39.4% of the population. Cell membrane damage increased by 38.4%, and 99.9% of the population exhibited mitochondrial depolarization (Figure 3D). No significant differences for intracellular ROS concentrations in epimastigotes subjected to normal nutritional conditions were observed between UBMC-4-treated and untreated parasites. However, parasites treated with the inhibitor and undergoing nutritional stress displayed a twofold ROS concentration as compared to untreated parasites (Figure 3E).

One of the clinically relevant forms of *T. cruzi* for Chagas disease are the trypomastigotes, the non-replicative mammalian host infective form where the effect of UBMC-4 was analyzed using apoptosis-like cell death markers and a comparison with epimastigotes was performed. In addition, two different variables related to this parasitic form that may involve changes in the action pattern of UBMC-4 were evaluated: the first is related to the hypothesis that trypomastigotes are constitutively under stressing conditions; i.e., being not replicative, this parasite form lives in low pH and scarce nutrients environments (for metacyclic trypomastigotes) under constant pressure from the immune system with drastic temperature changes (i.e., blood trypomastigotes). Trypomastigotes may be prone to activate constant mechanisms of tolerance to cellular stress; therefore, UBMC-4 might act regardless of the parasites being subjected to nutritional stress or not. The second is a hypothesis that has already been proven in different studies where trypomastigotes are shown to be highly autophagic, a process known to have a dual function inhibiting or promoting apoptosis-mediated cell death processes [37].

**Figure 4:**
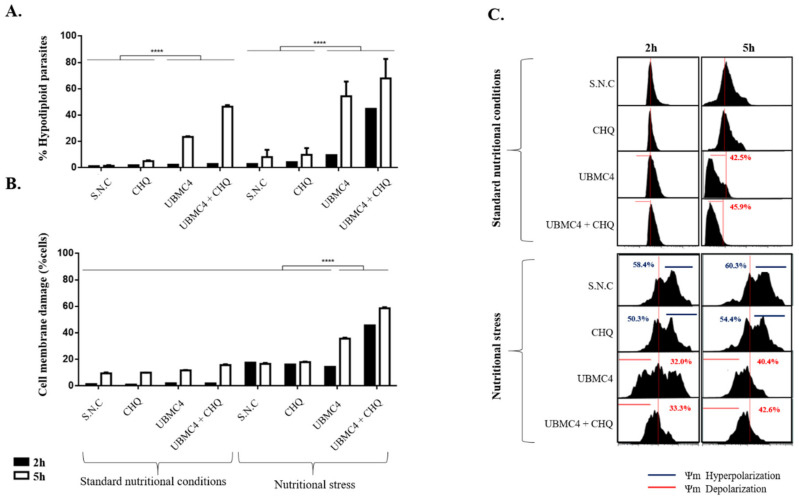
Determination of apoptosis-*like* in *T. cruzi* trypomastigotes undergoing nutritional stress and inhibition of AKT-like. The effects of AKT-like inhibition and autophagy were determined in *T. cruzi* trypomastigotes by flow cytometry. (**A**) Percentage of hypodiploidy in *T. cruzi* trypomastigotes under standard nutritional conditions and nutritional stress for 2 and 5 h. (**B**) Percentage of cell membrane damage in *T. cruzi* trypomastigotes under standard nutritional conditions and nutritional stress for 2 h and 5 h. (**C**) Frequency histogram of *Ψm* in *T. cruzi* trypomastigotes under standard nutritional conditions and nutritional stress for 2 h and 5 h. S.N.C, standard nutritional conditions; CHQ, chloroquine; S.C, conditions of nutritional stress. Asterisks indicate statistically significant differences regarding the values for control treatments (**** *p* < 0.0001).

In this sense, the inhibition of autophagy pathways through the use of a compound known as chloroquine could have a synergistic effect with respect to the effects by UBMC-4 on *T. cruzi* trypomastigotes [38]. The hypodiploidy marker revealed that UBMC-4 generated DNA damage in 54% of the trypomastigote population when they were subjected to conditions of nutritional stress (Figure 4A). However, despite the damage being less significant, UBMC-4 was able to promote DNA damage in 23% of the population of trypomastigotes cultured under normal nutritional conditions (Figure 4A), which supports the idea that these parasite forms are autophagic, unlike what occurs with epimastigotes. Interestingly, the combination of UBMC-4 with chloroquine promoted a synergy on the fragmentation of trypomastigote DNA under stress conditions, increasing this phenomenon given that, after 2 h of treatment, 44% of the parasite population experienced DNA damage, which increased to 68% after a 5 h treatment (Figure 4A). Similarly, the same synergistic effect between chloroquine and UBMC-4 was observed when trypomastigotes were subjected to normal nutritional conditions where 46% of the trypomastigotes population had fragmented DNA after 5 h of treatment, but no increased effect was observed since the damage did not exceed 5% after 2 h of exposure to both drugs under normal culture conditions (Figure 4A). 

Regarding cell membrane damage markers, we found that the parasites subjected to nutritional stress both at 2 h and 5 h displayed a percentage of permeability <20% (Figure 4B), as opposed to what was observed in trypomastigotes that were subjected to normal nutritional conditions where <2% and <10% permeability at 2 h and 5 h, respectively, was obtained (Figure 4B). 

UBMC-4, on the other hand, induced cell membrane permeability at 5 h of treatment under nutritional stress conditions where 36% of the parasite population suffered cell membrane permeability (Figure 4B). However, this was not a sustained effect under normal nutritional conditions where a cell membrane permeability of 2.2% was only achieved by UBMC-4 after 5 h of treatment compared to the untreated controls in the same time window (Figure 4B). On the other hand, if parasites were subjected to stress conditions, the same pattern of synergistic effect from chloroquine used along with UBMC-4 continued given that this combination treatment generated a cell membrane permeability of 45% after 2 h of treatment, while at 5 h a permeability of 58% was observed (Figure 4B). However, when both chloroquine and UBMC-4 were evaluated on parasites under normal nutritional conditions, no significant changes were observed at 2 h of treatment and only a 5.6% increase in permeability was observed at 5 h of treatment compared to untreated or chloroquine-only treated parasites (Figure 4B).

Regarding the analysis of changes in the mitochondrial membrane potential carried out at 2 h and 5 h under the same experimental conditions mentioned, we found that trypomastigotes subjected to nutritional stress tended to hyperpolarize the mitochondrial membrane in a progressive manner, comprising 58% at 2 h of treatment and 60% at 5 h of treatment, a phenomenon that is preserved with respect to what has been observed in *T. cruzi* epimastigotes (Figure 4C). Nonetheless, when trypomastigotes were subjected to normal nutritional conditions, a constant mitochondrial membrane potential was found and no significant changes in membrane hyper- or depolarization were found (Figure 4C). When the effect of UBMC-4 under nutritional stress conditions was tested, we found that stress promoted a progressive change of the mitochondrial membrane potential towards a depolarization status, beginning with 32% of parasites depolarized after 2 h of treatment and reaching 40% by 5 h (Figure 4C). 

This behavior seems not to be preserved when trypomastigotes are cultured under normal nutritional conditions, where the effects of UBMC-4 on the mitochondrial membrane potential were not evident. At 2 h of UBMC-4 treatment, the mitochondrial membrane potential of trypomastigotes remained intact (no hyper- or depolarization) while at 5 h the effect of UBMC-4 seemingly affected the parasites by depolarizing the mitochondrial membrane in 45% of the population (Figure 4C). If chloroquine alone was used as an inhibitor of autophagy processes, a steady mitochondrial membrane potential was found in trypomastigotes subjected to stress and normal nutritional conditions. Under stress conditions, the parasite membrane hyperpolarization continued just as in the untreated controls; under normal nutrition conditions, however, no alteration in the mitochondrial membrane potential of parasites was observed (Figure 4C).

When UBMC-4 and chloroquine were used in combined treatments, a constant depolarization pattern under conditions of nutritional stress was found, as was the case for the DNA damage marker (Figure 4A), although no marked effect was observed on the increased depolarization compared to the chloroquine-untreated, UBMC-4-only treated parasites (Figure 4C). Additionally, no significant changes in mitochondrial membrane depolarization were found when parasites were challenged with chloroquine and UBMC-4 under normal nutrition conditions for 2 h (Figure 4C). In spite of the effect observed under these same conditions at 5 h of treatment, resulting in a mitochondrial membrane depolarization of 42%, a phenomenon similar to what has been observed with the hypodiploidy marker where damage in the DNA under normal nutrition conditions was not enhanced by the use of both UBMC-4 and chloroquine at 2 h, an increased population of hypodiploid parasites was noted after 5 h of treatment (Figure 4A).

### 2.4. Plasma Concentration

The concentration results obtained by HPLC for each time point measured are shown in Table 1. Results show that the compound UBMC-4 is absorbed orally and UBMC-4 may yet be found in plasma at a concentration of 2.8 µg/mL (6.690 µM) 1 h after dose administration. In addition, the highest plasma concentration (C_max_) of 4.2 µg/mL (10.036 µM) was observed with a treatment of 3 h and UBMC-4 could still be found in the blood stream after 24 h at a concentration of 2.4 µg/mL (5.735 µM). Conversely, no peak other than that for the acetone used to treat such samples was shown in the chromatograms for samples collected from vehicle-treated mice (See Supplementary Materials).

### 2.5. Pharmacokinetic Modeling

As shown in Table 2, the best model for collecting plasma concentration data is a two-compartment model. A −Log-likelihood (−LL) of −100.874 and Akaike information criterion (AIC) of −187.749, the lowest values for the three models assessed, were obtained by this model. For the one-compartment and three-compartment models, the −LL and AIC values were not even negative, suggesting that the two-compartment model is more useful for pharmacokinetic analyses.

**Figure 5:**
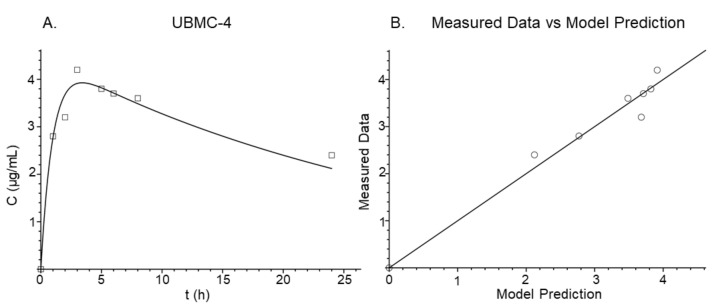
Two-compartment pharmacokinetic model adjusted for UBMC-4. Representation of the best adjusted pharmacokinetic model for UBMC-4 plasma concentrations after administration of a 200 mg/kg oral dose. Squares in panel (**A**) represent values measured in plasma whereas the solid line represents the values calculated using the model. Panel (**B**) shows the correlation between measured and predicted values.

The total clearance (CL_t_/F), central volume (V_c_/F), absorption constant (K_a_), intercompartmental clearance (CL_d_/F), and peripheral volume (V_p_/F) parameters estimated by the two-compartment model are shown in Table 3. Bioavailability (F) was not determined as intravenous administration data were not available. The estimated half-life (t1/2) was 22.38 h. For the final model, the standard deviation of the intercept was 0.2472 × 10^−44^ and the standard deviation of the slope was 0.1071. Furthermore, Figure 5A shows the adjusted model for the data obtained. Similarly, a correlation between the obtained results and the results predicted by the model was established (Figure 5B). For this correlation, the r^2^ was 0.968, demonstrating consistency between both predicted and observed data. The estimation of these parameters and consolidation of the model allowed the elaboration of simulations using different dosing schedules and collection of further information about the changes of plasma concentration profile after repeated dosing. 

### 2.6. Pharmacokinetic Simulation

**Figure 6:**
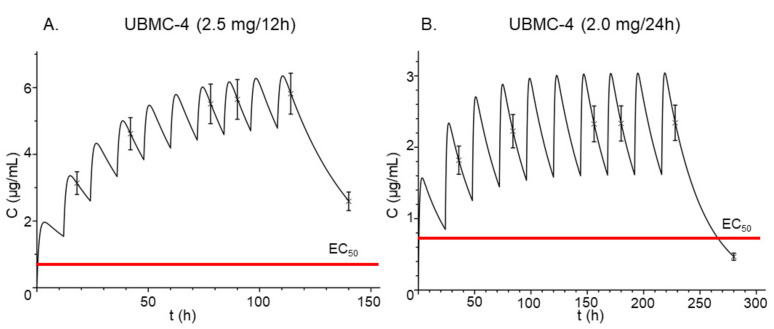
Pharmacokinetic simulation of UBMC-4. Simulation of UBMC-4 plasma concentrations over time after administration of multiple oral doses of (**A**) 2.5 mg every 12 h and (**B**) 2.0 mg every 24 h. Red line represents UBMC-4 in vitro half effective concentration, EC_50_, (0.774 µg/mL) on *T. cruzi*.

Once the two-compartment model was obtained, simulations using different dosing schedules were performed to understand the behavior of UBMC-4 after administration of repeated oral doses. Figure 6 shows plasma concentration levels after oral administration of (a) 2.5 mg every 12 h and (b) 2.0 mg every 24 h. In both cases, total clearance of UBMC-4 was not achieved between the doses administered, leading to an accumulation effect of the compound and consequently increased plasma concentrations. However, this effect was greater for the 2.5 mg regimen every 12 h. Application of both dosing schedules above resulted in plasma concentrations of UBMC-4 higher than the EC_50_ from the first dose and is shown in Figure 6 as well. Even if the dose was halved, a plasma concentration higher than the EC_50_ was achievable.

## 3. Discussion

*Trypanosoma cruzi* undergoes different types of stress during its complex life cycle and adaptability to these conditions in order to successfully infect, proliferate, and survive is required. In the present study, we showed that inhibition of the AKT-like kinase in *T. cruzi* induces loss of tolerance to nutritional stress, thereby promoting apoptosis-like events. Pre-existence of *T. cruzi* amastigotes within the tissue may affect a host for years or decades. Intercommunication between the parasite and cytosol components of parasitized cells is crucial for the spread and maintenance of the infection. However, little is known about how the parasite manages to survive for long periods without killing its host cell. *T. cruzi* promotes survival of its host cell through strategies such as production of proteins like the parasite-derived neurotrophic factor, a membrane surface trans-sialidase that induces AKT kinase activation in host cells [39]. Nevertheless, both the role of the AKT-like kinase and its participation in resistance to nutritional, immunological, and thermal stress events are unknown. A putative function has been extrapolated from that described in humans, where the AKT enzyme activates cell survival processes by inhibiting apoptosis and maintaining homeostasis [40]. 

Before genomic data became available, the AKT-like kinase of *T. cruzi* was first described as an enzyme with kinase activity and a putative PKB function. However, the biological function or importance as a molecular target was not described. Although previous research described that *T. cruzi* AKT-like, unlike human AKT kinases, lack the PH domain, this hypothesis was later rejected based on computational analysis [41]. The bioinformatics studies performed to elucidate the association between the PH domain of the AKT-like kinase in *T. cruzi* and the compound UBMC-4 suggest a potential interaction between the inhibitor and the PH domain of the enzyme. The obtained score for UBMC-4 predicted by AutoDock Vina was −8.3 kcal/mol, suggesting the likelihood of a protein-ligand interaction at the tested domain. Negative scores lower than −7 kcal/mol indicative of the affinity required for interaction have been reported in similar studies for other parasites [42,43]. Noteworthily, the score obtained is a prediction for the interaction of the ligand, specifically to the PH domain. This is interesting given that the PH domain in this parasite is the domain that displays the lowest percentage of identity as compared to human AKT. A more significant effect on parasites rather than in host cells could thus be favored, reducing the probability of exhibiting toxic effects on human cells [31]. However, further experimental studies on the interaction between UBMC-4 and the *T. cruzi* AKT-like recombinant protein are being performed where X-ray crystallography or NMR will be used to confirm the results from bioinformatics studies. 

Analysis of the effectiveness of UBMC-4 on intracellular amastigotes of *T. cruzi* (1.85 ± 1 μM) revealed a notably high activity against the parasite, where an EC_50_ very similar to the bz standard drug used (1.16 ± 0.03 μM) was reported. Besides, the EC_50_ obtained for bz in our study accorded with previous reports in the literature. Araujo-Lima et al. reported in 2018 an EC_50_ of 1.8 ± 0.7 μM against intracellular forms of Tulahuen strain [44]. The low toxicity of UBMC-4 on human cells and its high SI (21.6) should also be noted, therefore rendering favorability of UBMC-4 upon determination of the dose effective against the parasite and safe for the host. In addition, the efficacy on parasites and safeness for the host agree with the aforementioned results from the bioinformatics analyses. A non-reversible morphological damage in nutritionally stressed epimastigotes using a UBMC-4 concentration at least fivefold higher than the EC_50_ over 6 h was observed as well. These changes could be related to blockade of the PI3K/AKT/mTOR pathway, an event that may trigger apoptosis-like death in parasites [45]. 

Apoptosis markers were analyzed by flow cytometry after epimastigotes and trypomastigotes were subjected to nutritional stress and treated with the inhibitor UBMC-4, whereby gradual DNA fragmentation and membrane depolarization were associated with alterations of the mitochondrial membrane and increased generation of intracellular ROS. We interpreted those events as consequences of increased apoptosis-like conditions, revealing that AKT-like kinase is crucial for the response to nutritional stress in *T. cruzi* epimastigotes and trypomastigotes. For *T. cruzi* trypomastigotes, unlike the evidence found for epimastigotes, inhibition of AKT-like kinase in *T. cruzi* trypomastigotes under normal nutritional and nutritional stress conditions increased DNA fragmentation, mitochondrial depolarization, and cell membrane permeabilization, and caused much faster progressive morphological changes within the hour when incubated with 10 µM of UBMC-4 (data not shown), possibly due to the stress mechanisms activated by the parasite stage [31]. In addition, the use of 10 µM chloroquine, a classic autophagy inhibitor, accelerated and increased the effects of UBMC-4 with respect to the apoptosis-like markers evaluated. Activation of these events of the apoptosis cascade may indicate that UBMC-4 induces cell death phenotypes, depending on the environmental conditions and of *T. cruzi*’s life cycle stage [45]. 

These findings, in addition to the UBMC-4 plasma concentrations estimated by pharmacokinetic analysis, are highly valuable in predicting a potential trypanocidal effect for any specific dose of the compound in vivo. By using the pharmacokinetic model constructed, for instance, determination of a full dosing schedule whereby therapeutic effectiveness is expectable and achievement of a plasma concentration greater than the EC_50_ by administration of a fixed dose of UBMC-4 could be estimated [46,47]. For determination of UBMC-4 in mouse plasma, the HPLC method was considered suitable to quantify the compound in any sample analyzed and oral absorption of UBMC-4 was evidenced. For the HPLC method, an adequate linearity was achieved for the concentration range under analysis. In addition, upon analysis of samples from mice treated with UBMC-4 inhibitor-free vehicle, the specificity of the method was confirmed, as no additional peaks corresponding to different molecules interfering with the quantification of UBMC-4 were displayed in the chromatogram. Regarding plasma concentrations, we observed that UBMC-4 was already circulating in blood at a concentration of 2.8 µg/mL after 1 h. The fact that UBMC-4 is absorbed orally is important if dosing to humans is to be considered, as the oral route is the most accepted administration route. The highest UBMC-4 concentration of 4.5 µg/mL was reported after 3 h; however, rapid clearance was not observed and after 24 h a significant UBMC-4 concentration of 2.4 µg/mL was still detectable in systemic circulation, which is in agreement with the obtained half-life, a pattern that was adequately described by the two-compartment model (Figure 5). Of note, this concentration quantified in plasma should be taken into account as it is not necessarily the same at the intracellular milieu where amastigotes are hosted. For other antibiotics, however, a correlation between this concentration and the therapeutic efficacy even against intracellular microorganisms has been observed [36,47,48]. On the other hand, bloodstream circulating trypomastigotes will indeed be directly exposed to the concentration measured by HPLC, which would be lethal for the parasite as shown in the SEM test.

The values (squares) in Figure 5A show closeness to those predicted by the model. The major deviation of the predicted values as compared to the observed values was on Cmax 3 h after administration. Despite that, a suitable r^2^ value was obtained when a correlation of the observed values was estimated and those values were plotted as opposed to the predicted values (Figure 5, panel B), thus suggesting that the model is useful for predicting UBMC-4 concentration values at different time points. Considering this, simulations were performed using different doses and repeated doses (Figure 6). 

Upon administration of repeated doses even every 24 h (Figure 6, Panel B), the compound accumulated, reaching concentrations much higher than the EC_50_, which is represented by the red line plotted in both panels of Figure 6. Accumulation of the compound may lead to concentrations up to sevenfold the EC_50_ for the 2.5 mg/12 h regimen and fourfold the EC_50_ for the 2.0 mg/24 h regimen for a 10-day sham treatment. However, in a longer treatment, even higher concentrations will be reached. For the 2.5 mg/12 h regimen, it could lead to unnecessarily high concentrations for the treatment of trypanosomiasis in mouse models. Therefore, a dosing regimen using 2.0 mg/24 h is recommended to perform the analysis of efficacy. Moreover, when conducting efficacy testing in mice a special consideration should be given to any potential differences between the plasma concentration profiles of UBMC-4 resulting from the physiological changes caused by the *T. cruzi* infection. For this assay, maintaining a UBMC-4 plasma concentration at least fourfold the EC_50_ throughout the treatment would be ideal.

In the pipeline of developing a new drug to treat an infectious disease, the target product profile (TPP) must always be kept in mind. In the case of Chagas disease, the Drugs for Neglected Diseases *initiative* (DND*i*) has compiled the TPP that should present a new treatment for the disease [49]. UBMC-4 has some desirable characteristics that point it out as a promising candidate that meets some of the guidelines established in the TPP. This compound is active on trypomastigotes that circulate in the blood and can be associated with the acute form of the disease, but activity was also observed on intracellular amastigotes that can be related to the chronic form [50]. In addition, the activity presented on amastigotes is comparable with that of the reference drug benznidazole. On the other hand, according to the TPP, an advantage of UBMC-4 found in this study is the possibility of oral administration. Pharmacokinetic tests showed that oral administration allows sufficient concentrations to kill the parasite. These findings should be complemented with future studies where the intravenous route is evaluated. In this way, the total bioavailability of the compound can be determined, which according to the general criteria presented in the work of Katsuno et al. should be greater than 25% [51]. Regarding the physicochemical properties, UMBC-4 presents slight solubility problems. However, with the formulation developed, the compound was able to be dissolved and was administered as an oral solution. In addition, it did not exhibit acute toxicity at the dose tested and did not cause death to any of the mice in the pharmacokinetic assay [49,50,51]. Finally, the toxicity and effectiveness of UMBC-4 should be evaluated in vivo to see whether they fit the TPP defined for new candidates for the treatment of Chagas disease. Hence, in the development process of the UBMC-4 compound, the next step should be the determination of the half-lethal concentration LC_50_ in vivo and evaluating its effectiveness in an in vivo model of Chagas disease.

## 4. Materials and Methods

### 4.1. Molecular Modeling of T. cruzi AKT-like Protein Structure and UBMC-4 Molecular Docking

Due to the unavailability of an experimentally-solved 3D structure, the amino acid sequence of the *T. cruzi* AKT-like protein (UniProtKB id Q4D6D3) was used as input in a threading approach on the I-TASSER web server [52,53]. The potential structure of the enzyme with the pleckstrin homology domain (PH) was submitted to quality testing through various metrics derived from the Ramachandran plot, as well as energy curves calculated in the SwissModel server [54]. Finally, the protein was minimized with gradient descent using the minimization protocol available in UCSF Chimera version 1.10 [55]. The 3D structure of UBMC-4 was downloaded from the ZINC database [56]. Favorable predicted physicochemical properties according to the drug-like filters proposed in former studies were reported for both compounds [29,57]. The protein structure model was submitted to the Ligsite web-server, where a list of potential binding sites was predicted [58]. In our case, we selected the region surrounding the PH domain and both the protein and compound were subsequently parameterized using AutoDock Tools [59]. This process involves adding hydrogen bonds to the polar side chains and estimating the partial charges using the Gasteiger methodology. In the docking strategy, the compounds were considered flexible based on the active torsion bonds of 3D structures. The docking protocol was further executed in AutoDock Vina [60] using 20 internal replicates per molecule and scores were estimated in kcal/mol, whereas the number and type of molecular interactions was inspected by plots.

### 4.2. Chemicals

Ambiter UBMC-4 (Amb16761662): 4-(4-dibenzo-[b,d]-thien-2-yl-5-phenyl-1H-imidazol-2-yl)phenol, Basf Kolliphor RH40, Acetone, Acetonitrile, Methanol (Merck, Rahway, NJ, USA).

### 4.3. Cell Lines and Parasite Strains

All cell lines were from ATCC (American Type Cell Culture, Mannassas, VA, USA). VERO cells (ATCC CCL-81), HepG2 human liver cells (ATCC HB-8065), and CaCo2 human epithelial colorectal carcinoma cells (ATCC HTB-37) were cultured in 5% FBS, 1% antibiotics (100 U/mL penicillin and 0.1 mg/mL streptomycin) DMEM medium. 

In turn, the promonocytic cell line U937 (ATCC CRL-1593.2) was maintained in RPMI 1640 containing 10% FBS, 2 mM 1-glutamine, and 1% antibiotics. U937 cells were differentiated by seeding at a starting cell concentration of 1 × 10^6^ cells/mL in a medium containing 100 nM phorbol 12-myristate 13-acetate (PMA) for 48 h. All cell cultures were kept under the same conditions at 37 °C and a 5% CO_2_ atmosphere. The cells were then adherent and slightly flattened. In addition, huMDM were obtained as described by Daigneault and collaborators [61]. 

*T. cruzi* epimastigotes from Gal61 (MRAT/COL/Gal61, donated by Dr. Ana Maria Mejia from the BCEI laboratory of the University of Antioquia) and Tulahuen (ATCC 30266) strains transfected with the *T. cruzi* β-galactosidase gene were used for SEM experiments and trypanocidal activity, respectively. The Gal61 epimastigotes were cultured at 28 °C in RMPI 1640 medium supplemented with 0.02% hemin, 0.5% trypticase peptone, 0.002 M HEPES, 10% inactivated FBS, and 1% antibiotics. Tulahuen epimastigotes were cultured in a modified NNN medium with liquid phase (8.5 NaCl; 10 g glucose; 1 L MQ H20) at 26 °C [62]. Parasites were subcultured on a weekly basis [49,50].

To obtain trypomastigotes, 2 × 10^6^ VERO cells were cultured in 10% FBS-supplemented DMEM medium at 37 °C and 5% CO_2_ until reaching 90% of confluence. Subsequently, the 10% FBS supplemented DMEM medium was replaced by FBS-free DMEM medium to obtain a no growing monolayer for further infection using epimastigotes (Gal61 strain) in exponential growth phase at a parasite:VERO cell ratio of 3:1. Cells and epimastigotes were incubated at 37 °C with 5% CO_2_ in the FBS-free DMEM medium. Culture-derived trypomastigotes were harvested after 10 to 12 days of infecting VERO cells.

A total of 1×10^6^ trypomastigotes from FBS-free VERO cells were harvested and centrifuged at 2000 rpm for 5 min and the culture medium was removed. Subsequently, parasites were reconstituted in PBS to generate conditions of nutritional stress. 

### 4.4. Cytotoxicity 

Cytotoxicity of UBMC-4 was tested in HepG2, CaCo2, and huMDM cell lines according to the effect on cell growth determined by the MTT microenzimatic method, as described by others [63]. Briefly, 2.0 × 10^4^ U-937 and huMDM or 2.5 × 10^4^ HepG2 and CaCo2 cells were dispensed in 200 µL into each well of a 96-well tissue culture plate containing the corresponding culture medium. Then, 100 µL/well of each compound at a concentration of 200, 100, 50, 25, 12.5, or 6.25 µL/mL were added and plates were incubated at 37 °C, 5% CO_2_. After 72 h of incubation, 20 μL of MTT (Sigma-Aldrich, St Louis, MO, USA) were added to each well and plates were incubated for 3 h at 37 °C, 5% CO_2_. Reaction was stopped by adding 100 μL/well of dimethyl sulfoxide (DMSO) to each well and 30 min of incubation. The concentration of formazan was determined at 570 nm in a spectrophotometer (Varioskan Flash Multimode Reader, Thermo Scientific, Waltham, MA, USA). Cells treated with benznidazole (bz) as the standard anti-trypanosomal drug and doxorubicin were used as a control for cytotoxicity (positive control) while cells incubated in absence of any compound or drug were used as control for cell growth (negative control). Determinations were done by triplicate in at least two independent experiments. Cytotoxicity was determined according to the percentages of viability and cell growth inhibition obtained for each compound, bz, doxorubicin, or medium alone. Non-specific absorbance was corrected by subtracting the OD of the blank (culture medium). Percentages of mortality were calculated using equation, as follows: % mortality = 100 − [(O.D. of treated cells)/(O.D. of non-treated cells)] × 100. The half maximal effective concentration (EC_50_) was calculated by the linear regression model Probit using Graphpad Prism software.

### 4.5. Trypanocidal Activity of UBMC-4 in Intracellular Amastigotes

To obtain intracellular amastigotes, 2.5 × 10^6^ U937 differentiated macrophage cells were infected with *T. cruzi* epimastigotes (Tulahuen strain producing β-galactosidase, 24 h of growing) in a 5:1 parasite/cell ratio. To calculate EC_50_, 100 μL of UBMC-4 were added at four serial dilutions (50, 12.5, 3.12, and 0.78 μg/μL) to each well, and benznidazole (bz) was added at an initial concentration of 20 µg/mL and 6 serial 2-fold dilutions were used to determine its EC_50_ as a control of the effectiveness of the experiment, in addition to the control of the viability of the parasites (cells without drug treatment), from the blank of the culture medium (medium without cells), in duplicate. The cells were incubated for 72 h at 37 °C with 5% of CO_2_. All medium was removed and 100 µL of substrate for β-galactosidase diluted in PBS (chlorophenol β-d-galactopyranoside-CPRG network; Sigma-Aldrich, St Louis, MO, USA, at 100 mM and 0.1% Nonidet P-40) was added to each well and incubated at 37 °C for 3 h. The amount of chlorophenol red released was read at 570 nm in a spectrophotometer (Varioskan TM). Infected cells were used as negative control while infected cells treated with benznidazole were used as a positive control (trypanocidal activity) for the assay. RPMI-1640 medium was used as a negative control. Non-specific absorbance was corrected by subtracting the OD of the blank sample. Determinations were done in triplicate in at least two independent experiments.

The trypanocidal activity was determined based on the percentage of decrease of amastigotes determined using equation, as follows: % reduction of infection = 100 − (O.D. of treated infected cells)/(O.D. of non-treated infected cells) × 100. The half maximal effective concentration (EC_50_) was calculated by the linear regression model Probit using Graphpad Prism software.

### 4.6. Scanning and Transmission Electron Microscopy 

Epimastigotes untreated and treated with 10 uM of UBMC-4 under nutritional stress conditions (culture medium substitution for PBS for 6 h) were fixed for 2 h using 2.5% glutaraldehyde in cacodylate buffer 0.1 M. Parasites adhered for 10 min onto coverslips previously coated with 0.1% poly-L-lysin and subsequently washed with cacodylate buffer and dehydrated using 30%, 50%, 70%, and 90% (*v*/*v*) acetone for 5 min on each incubation. Subsequently, the cells were critically dried under CO_2_ and attached to the heel of the SEM instrument. Finally, samples were coated with a 20 nm-thick gold layer and analyzed in a Jeol JSM6010Plus-LA scanning electron microscope at 20 kV. For TEM, parasites were fixed for 2 h with glutaraldehyde and cacodylate buffer and then treated for 1 h with 1% OsO4 diluted in the same buffer. Parasites were dehydrated as above and then embedded in PolyBed-812 resin. Ultrathin sections were collected in copper grids and stained with 5% aqueous uranyl acetate and lead citrate. Samples were examined in a Jeol JEM-1400plus transmission electron microscope at 80 kV.

### 4.7. Flow Cytometry Analysis of Apoptosis-like Markers in T. cruzi

Apoptosis-like markers were analyzed by triple staining using 3,3′-dihexyloxacarbocyanine (DiOC6), propidium iodide (PI), and Hoechst from Thermo Fisher Scientific to determine the change of mitochondrial membrane potential (Δ*Ψm*), cell membrane damage, and impairment of cell cycle, respectively [64]. All readings were performed on a BD FACSCanto II 4/2/2 Sys IVD flow cytometer, and data obtained were analyzed using the FlowJo7.6.2 software. Differences between treatments were determined by a two-way ANOVA for multiple comparisons with 95% significance using GraphPad Prism 8. In all cases, 20 µL of parasites (5 × 10^6^/mL) were stained per treatment with 300 µL of Hanks’ balanced saline solution (HBSS) and 10 µL of the staining mix containing 10 µL of 5 μg/mL PI, 0.5 µL of 70 nM DiOC6, and 0.5 µL of 10 mg/mL Hoechst. Subsequently, parasites were incubated at room temperature for 15 min until reading.

*T. cruzi* epimastigotes in exponential growth phase were used to submit the parasites to a treatment under nutritional stress and further challenging the culture with the inhibitor UBMC-4 and the control drug bz. Nutritional stress was induced by starving the parasites in PBS. Parasites were grown either in complete RPMI medium or PBS (with and without 10 µM UBMC-4) for 24 h at 28 °C. Three independent biological replicates were analyzed per treatment. The experiments were performed in 24-well plates at an initial concentration of 1 × 10^7^ total parasites in 2 mL/well. Parasites were centrifuged at 1800 RCF for 5 min, washed once with PBS buffer, and then resuspended in a final volume of 2 mL for further labeling and flow cytometry.

The same markers mentioned above were used for *T. cruzi* trypomastigotes. A total of 1 × 10^6^ trypomastigotes were centrifuged at 1800 RCF for 5 min to remove the culture medium. Parasites were cultured at 3 °C in PBS or 10% FBS-supplemented DMEM with or without 10 µM UBMC-4, 10 µM chloroquine, and 10 µM UBMC-4/chloroquine for 2 h and 5 h. All experiments were compared to a culture setup using bz as control compound. Three biological replicates and three experimental replicates were applied to data collected at each time point for experimental conduction of every portion of the study. In all cases, ANOVA was applied for multiple comparisons after determining that data were normal. A Kruskal–Wallis statistical test was applied if data showed a non-parametric distribution.

### 4.8. Analysis of Intracellular Production of Reactive Oxygen Species (ROS) in UBMC-4-Treated T. cruzi Epimastigotes

A total of 1.4 × 106 epimastigotes grown under normal and stress nutritional conditions were treated with 10 µM UBMC-4 for 24 h and the intracellular ROS concentration was determined by fluorometric testing with the diacetate 2′,7′-dichlorodihydrofluorescein (H2DCFDA) probe at 0.2 µg/mL. As controls, cell cultures both treated with no probe as well as only labeled with H2DCFDA were performed to determine baseline fluorescence. A ROS-production positive control was set up by challenging parasites with 50 mg/mL rotenone (a ROS inducer) for 1 h prior to labeling and reading. After the treatment, parasites were washed and resuspended in 100 µL of the H2DCFDA-containing solution, transferred to 96-well plates protected from the light, and then incubated for 1 h at 37 °C. The test readout was collected using an ELISA plate reader at an excitation wavelength of 495 nm and emission wavelength of 527 nm, respectively. Each treatment was performed in triplicate, and data were reported in arbitrary units of fluorescence [65].

### 4.9. Formulation

Kolliphor RH40 (0.9 g) was brought to a temperature of 50–60 °C and 50 mg of UBMC-4 were added. The preparation was shaken vigorously and subjected to ultrasound until full dissolution. After this, water previously heated at 50–60 °C was poured into the kolliphor-UBMC-4 solution under constant shaking. The same procedure was followed to prepare the vehicle using the same quantities but without adding UBMC-4.

### 4.10. Animals

The Balb/c endogamic strain of the *Mus musculus* specific-pathogen-free murine species was used. Twelve-week-old females and males were used. Mice were kept at 22–23 °C, 50–60% relative humidity, 16–20 air changes per hour regulated with a ventilation system and white light artificial illumination in a timer-controlled, 12:12 h light-dark cycle. Same sex, four-animal matched groups were arranged and housed into 19 cm H × 20 cm W × 30 cm L propylene boxes. Two-thirds of each box was filled with autoclave sterilized pine tree shavings. Mice received previously sterilized water and rodent food (LabDiet^®^). The use of experimental animals was approved by the Universidad de Antioquia Animal Research Ethics Committee (CEEA) pursuant to Record no. 110 on 17 May 2017.

### 4.11. In Vivo Pharmacokinetic Analysis of UBMC-4 

A total of 30 mice (15 males and 15 females) was randomly selected and divided into two groups: one group received a 200 µL oral single dose of UBMC-4 at a concentration of 60 mM (equivalent to a 200 mg/kg dose), and a control group receiving 200 µL of vehicle (Kolliphor and water without UBMC-4) via oral route. Considering that the average weight of the mice was 25 g, the total amount of UBMC-4 administered was 5 mg. The compound was only administered orally, it was not used intravenously since one of the primary objectives was to evaluate its gastrointestinal absorption. The dose was selected considering a preliminary toxicity study where significant tolerability of the compound was reported at 200 mg/kg administered once and 100 mg/kg every 24 h for 30 days. In this way, we wanted to find the highest potential plasma concentrations before inducing toxicity. Blood samples were collected in EDTA tubes via intracardiac puncture performed under general anesthesia in both groups at sampling time points as follows: 0 h, 1 h, 2 h, 3 h, 5 h, 6 h, 8 h, and 24 h. Samples were then centrifuged at 1800 rpm at 4 °C for 10 min to collect plasma. For each sampling time point, 100 µL of mouse plasma were collected and 200 µL of acetone were added. This mixture was vortexed for 30 s and centrifuged at 10,000 rpm at 4 °C for 20 min. Supernatants were poured into vials for HPLC analysis following the method described below.

The amount of UBMC-4 in plasma samples was quantified by a HPLC method using a Hitachi Chromaster HPLC System with a Knauer C18 Column as follows: λ: 300 nm; temperature: 40 °C; run time: 13 min at a flow rate of 1.0 mL/min. A gradient of acetonitrile:water:methanol in a 30:40:30 ratio was used as mobile phase from baseline to minute 6, a 20:0:80 ratio from minute 7 to minute 10, and again a 30:40:30 ratio from minute 11. Linearity of the method was calculated for the 0.5 µg/mL to 20 µg/mL concentration range, with a r^2^ (correlation coefficient) of 0.969 for the relationship between the area under the curve and UBMC-4 absorption at 300 nm. 

The ADAPT 5 software was used to determine the most suitable model for the UBMC-4 plasma concentration profile [66]. The maximum likelihood estimation method was used for modeling. After conducting trials where various models were used, the best one was chosen based on the -Log-Likelihood (-LL) and Akaike information criterion (AIC) values [67]. Consequently, by comparing models, the one producing lower values amongst these two parameters would be the one that best fit the plasma concentration data and the most useful for pharmacokinetic studies. Moreover, based on the R-square value (r^2^), we assessed the correlation between the values predicted by the model and those observed in UBMC-4 plasma concentration. The selected model was therefore used to elaborate simulations using different administration regimens and thus predicting what would happen with UBMC-4 concentration levels upon administration of repeated dosage. This simulation was also performed using ADAPT 5 [36,66,67]

## 5. Conclusions

We found that UBMC-4 has a strong activity in vitro (1.85 ± 1 μM) with an EC_50_ very similar to that of the standard drug benznidazole. We also observed morphological damage in parasites after 6 h of treatment with UBMC-4 which could be associated with the induction of a death phenotype such as apoptosis. Furthermore, pharmacokinetic modeling and simulation showed that oral dosing is an effective administration route because UBMC-4 can be found in plasma 1 h after the oral dose is given. In addition, we predict that UBMC-4 can reach plasma concentrations higher than the experimental EC_50_ concentrations. All collected data suggest that UBMC-4 is a potential candidate to carry out in vivo assays and a promising compound to treat Chagas disease.

## 6. Patents

The compound UBMC-4 was patented by our research group and resolution 45984 grants the patent under file reference NC2017/0000871 at the Superintendence of Industry and Commerce of the Republic of Colombia.

## Figures and Tables

**Table 1:** Plasma concentrations of UBMC-4 over time.

Time (h)	Concentration (μg/mL)
0	0
1	2.8
2	3.2
3	4.2
5	3.8
6	3.7
8	3.6
24	2.4

**Table 2:** Pharmacokinetic models tested and selection parameters.

Selection Parameters	One-Compartment	Two-Compartment	Three-Compartment
−Log-Likelihood (−LL)	14.903	−100.874	8.636
Akaike Information Criterion (AIC)	41.806	−187.749	33.372
r^2^	0.737	0.968	0.882

**Table 3:** Parameters estimated for the two-compartment model following the administration of a 200 mg/kg dose of UBMC-4.

Parameter	Estimated Value
CL_t_/F(L/h)	0.0357
V_c_/F (L)	0.6930
K_a_ (h^−1^)	0.6213
CL_d_/F (L/h)	0.2514
V_p_/F (L)	0.4360

## Data Availability

The datasets used and analyzed for quantification of the compound and pharmacokinetic modeling during this study are included as a supplementary information file.

## Supplementary Materials

The Supplementary Materials are available online at https://www.mdpi.com/article/10.3390/pathogens11060616/s1. Figure S1: Absorption spectrum of UBMC-4; Table S1: Gradient HPLC method; Table S2: Calibration curve for UBMC-4; Figure S2: Calibration curve for UBMC-4; Figure S3: HPLC method selectivity.

## References

[1] Liu Q., Chen J., Zhou X.-N. (2020). Preparedness for Chagas disease spreading worldwide. Infect. Dis. Poverty.

[2] Lidani K.C.F., Andrade F.A., Bavia L., Damasceno F.S., Beltrame M.H., Messias-Reason I.J., Sandri T.L. (2019). Chagas Disease: From Discovery to a Worldwide Health Problem. Front. Public Health.

[3] Junior P.A.S., Molina I., Murta S.M.F., Sánchez-Montalvá A., Salvador F., Correa-Oliveira R., Carneiro C.M. (2017). Experimental and Clinical Treatment of Chagas Disease: A Review. Am. J. Trop. Med. Hyg..

[4] Müller Kratz J., Garcia Bournissen F., Forsyth C.J., Sosa-Estani S. (2018). Clinical and pharmacological profile of benznidazole for treatment of Chagas disease. Expert Rev. Clin. Pharmacol..

[5] Altcheh J., Moscatelli G., Moroni S., Garcia-Bournissen F., Freilij H. (2011). Adverse Events After the Use of Benznidazole in Infants and Children With Chagas Disease. Pediatrics.

[6] Hasslocher-Moreno A.M., do Brasil P.E.A.A., de Sousa A.S., Xavier S.S., Chambela M.C., Sperandio da Silva G.M. (2012). Safety of benznidazole use in the treatment of chronic Chagas’ disease. J. Antimicrob. Chemother..

[7] Tornheim J.A., Beltran D.F.L., Gilman R.H., Castellon M., Mercado M.A.S., Sullca W., Torrico F., Bern C. (2013). Improved Completion Rates and Characterization of Drug Reactions with an Intensive Chagas Disease Treatment Program in Rural Bolivia. PLoS Negl. Trop. Dis..

[8] Jackson Y., Wyssa B., Chappuis F. (2020). Tolerance to nifurtimox and benznidazole in adult patients with chronic Chagas’ disease. J. Antimicrob. Chemother..

[9] Maguire J.H. (2006). Chagas’ disease—can we stop the deaths?. N. Engl. J. Med..

[10] Urbina J.A. (2015). Recent clinical trials for the etiological treatment of chronic chagas disease: Advances, challenges and perspectives. J. Eukaryot. Microbiol..

[11] Molina-Morant D., Fernández M.L., Bosch-Nicolau P., Sulleiro E., Bangher M., Salvador F., Sanchez-Montalva A., Ribeiro A.L.P., de Paula A.M.B., Eloi S. (2020). Efficacy and safety assessment of different dosage of benznidazol for the treatment of Chagas disease in chronic phase in adults (MULTIBENZ study): Study protocol for a multicenter randomized Phase II non-inferiority clinical trial. Trials.

[12] Martínez-Peinado N., Cortes-Serra N., Losada-Galvan I., Alonso-Vega C., Urbina J.A., Rodríguez A., VandeBerg J.L., Pinazo M.-J., Gascon J., Alonso-Padilla J. (2020). Emerging agents for the treatment of Chagas disease: What is in the preclinical and clinical development pipeline?. Expert Opin. Investig. Drugs.

[13] Alonso-Padilla J., Cortés-Serra N., Pinazo M.J., Bottazzi M.E., Abril M., Barreira F., Sosa-Estani S., Hotez P.J., Gascón J. (2019). Strategies to enhance access to diagnosis and treatment for Chagas disease patients in Latin America. Expert Rev. Anti. Infect. Ther..

[14] Field M.C., Horn D., Fairlamb A.H., Ferguson M.A.J., Gray D.W., Read K.D., De Rycker M., Torrie L.S., Wyatt P.G., Wyllie S. (2017). Anti-trypanosomatid drug discovery: An ongoing challenge and a continuing need. Nat. Rev. Microbiol..

[15] De Koning H.P. (2017). Drug resistance in protozoan parasites. Emerg. Top. life Sci..

[16] Khare S., Nagle A.S., Biggart A., Lai Y.H., Liang F., Davis L.C., Barnes S.W., Mathison C.J.N., Myburgh E., Gao M.-Y. (2016). Proteasome inhibition for treatment of leishmaniasis, Chagas disease and sleeping sickness. Nature.

[17] Alvarez V.E., Iribarren P.A., Niemirowicz G.T., Cazzulo J.J. (2021). Update on relevant trypanosome peptidases: Validated targets and future challenges. Biochim. Biophys. Acta—Proteins Proteom..

[18] Beltrán-Hortelano I., Perez-Silanes S., Galiano S. (2017). Trypanothione Reductase and Superoxide Dismutase as Current Drug Targets for Trypanosoma cruzi: An Overview of Compounds with Activity against Chagas Disease. Curr. Med. Chem..

[19] Matoba K., Nara T., Aoki T., Honma T., Tanaka A., Inoue M., Matsuoka S., Inaoka D.K., Kita K., Harada S. (2009). Crystallization and preliminary X-ray analysis of aspartate transcarbamoylase from the parasitic protist {\it Trypanosoma cruzi}. Acta Crystallogr. Sect. F.

[20] Trevisan R.O., Santos M.M., Desidério C.S., Alves L.G., Sousa T.D.J., Oliveira L.D.C., Jaiswal A.K., Tiwari S., Bovi W.G., De Oliveira-Silva M. (2020). In Silico Identification of New Targets for Diagnosis, Vaccine, and Drug Candidates against Trypanosoma cruzi. Dis. Markers.

[21] Lima C.R., Carels N., Guimaraes A.C.R., Tufféry P., Derreumaux P. (2016). In silico structural characterization of protein targets for drug development against Trypanosoma cruzi. J. Mol. Model..

[22] Capriles P.V.S.Z., Guimarães A.C.R., Otto T.D., Miranda A.B., Dardenne L.E., Degrave W.M. (2010). Structural modelling and comparative analysis of homologous, analogous and specific proteins from Trypanosoma cruzi versus Homo sapiens: Putative drug targets for chagas’ disease treatment. BMC Genom..

[23] Izumi E., Ueda-Nakamura T., Dias Filho B.P., Veiga Júnior V.F., Nakamura C.V. (2011). Natural products and Chagas{’} disease: A review of plant compounds studied for activity against Trypanosoma cruzi. Nat. Prod. Rep..

[24] Pereira R.M., Greco G.M.Z., Moreira A.M., Chagas P.F., Caldas I.V.O.S., Gonçalves R.V., Novaes R.D. (2017). Applicability of plant-based products in the treatment of Trypanosoma cruzi and Trypanosoma brucei infections: A systematic review of preclinical in vivo evidence. Parasitology.

[25] Jones A.J., Grkovic T., Sykes M.L., Avery V.M. (2013). Trypanocidal Activity of Marine Natural Products. Mar. Drugs.

[26] Torrico F., Gascón J., Barreira F., Blum B., Almeida I.C., Alonso-Vega C., Barboza T., Bilbe G., Correia E., Garcia W. (2021). New regimens of benznidazole monotherapy and in combination with fosravuconazole for treatment of Chagas disease (BENDITA): A phase 2, double-blind, randomised trial. Lancet Infect. Dis..

[27] Gashaw I., Ellinghaus P., Sommer A., Asadullah K. (2012). What makes a good drug target?. Drug Discov. Today.

[28] Varela R.E.M., Ochoa R., Muskus C.E., Muro A., Mollinedo F. (2017). Identification of a RAC/AKT-like gene in Leishmania parasites as a putative therapeutic target in leishmaniasis. Parasites Vectors.

[29] Tirado-Duarte D., Marín-Villa M., Ochoa R., Blandón-Fuentes G., Soares M.J., Robledo S.M., Varela-Miranda R.E. (2018). The Akt-like kinase of Leishmania panamensis: As a new molecular target for drug discovery. Acta Trop..

[30] Ochoa R., Rocha-Roa C., Marín-Villa M., Robledo S.M., Varela-M R.E. (2018). Search of allosteric inhibitors and associated proteins of an AKT-like kinase from trypanosoma cruzi. Int. J. Mol. Sci..

[31] Díez A.F. (2019). Caracterización Funcional de la Quinasa AKT-like de Trypanosoma cruzi y Trypanosoma Brucei como Blanco para una Nueva Estrategia Terapéutica Contra la Tripanosomiasis. Master’s Thesis.

[32] Hanada M., Feng J., Hemmings B.A. (2004). Structure, regulation and function of PKB/AKT—A major therapeutic target. Biochim. Biophys. Acta—Proteins Proteom..

[33] Gross S., Rahal R., Stransky N., Lengauer C., Hoeflich K.P. (2015). Targeting cancer with kinase inhibitors. J. Clin. Investig..

[34] Lindsley C.W., Barnett S.F., Yaroschak M., Bilodeau M.T., Layton M.E. (2007). Recent progress in the development of ATP-competitive and allosteric Akt kinase inhibitors. Curr. Top. Med. Chem..

[35] Digirolamo F., Miranda M., Bouvier L., Cámara M., Cánepa G., Claudio Pereira La Vía de Transducción de Señales tor de Mamíferos Está Presente en Trypanosoma Cruzi (2012). Reconstrucción In Silico y Posibles Funciones. Medicina (Buenos Aires).

[36] Bonate P.L. (2011). Pharmacokinetic-Pharmacodynamic Modeling and Simulation.

[37] Menna-Barreto R.F.S. Between Armour and Weapons—Cell Death Mechanisms in Trypanosomatid Parasites. https://www.intechopen.com/chapters/48836.

[38] Kimura T., Takabatake Y., Takahashi A., Isaka Y. (2013). Chloroquine in cancer therapy: A double-edged sword of autophagy. Cancer Res..

[39] Chuenkova M.V., PereiraPerrin M. (2009). Trypanosoma cruzi Targets Akt in Host Cells as an Intracellular Antiapoptotic Strategy. Sci. Signal..

[40] Manning B.D., Toker A. (2017). AKT/PKB Signaling: Navigating the Network. Cell.

[41] Pascuccelli V., Labriola C., Téllez-Iñón M.T., Parodi A.J. (1999). Molecular and biochemical characterization of a protein kinase B from Trypanosoma cruzi. Mol. Biochem. Parasitol..

[42] Adejoro I.A., Waheed S.O., Adeboye O.O. (2016). Molecular Docking Studies of Lonchocarpus cyanescens Triterpenoids as Inhibitors for Malaria. J. Phys. Chem. Biophys..

[43] Dhorajiwala T.M., Halder S.T., Samant L. (2019). Comparative in silico molecular docking analysis of l-threonine-3-dehydrogenase, a protein target against African trypanosomiasis using selected phytochemicals. J. Appl. Biotechnol. Rep..

[44] Araujo-Lima C.F., Peres R.B., Silva P.B., Batista M.M., Aiub C.A.F., Felzenszwalb I., Soeiro M.N.C. (2018). Repurposing Strategy of Atorvastatin against Trypanosoma cruzi: In Vitro Monotherapy and Combined Therapy with Benznidazole Exhibit Synergistic Trypanocidal Activity. Antimicrob. Agents Chemother..

[45] Menna-Barreto R.F.S. (2019). Cell death pathways in pathogenic trypanosomatids: Lessons of (over)kill. Cell Death Dis..

[46] Angus B.J., Thaiaporn I., Chanthapadith K., Suputtamongkol Y., White N.J. (2002). Oral artesunate dose-response relationship in acute falciparum malaria. Antimicrob. Agents Chemother..

[47] Kaiser M., Bray M.A., Cal M., Trunz B.B., Torreele E., Brun R. (2011). Antitrypanosomal activity of fexinidazole, a new oral nitroimidazole drug candidate for treatment of sleeping sickness. Antimicrob. Agents Chemother..

[48] Levêque D., Becker G., Bilger K., Natarajan-Amé S. (2020). Clinical Pharmacokinetics and Pharmacodynamics of Dasatinib. Clin. Pharmacokinet..

[49] DNDi—Drugs for Neglected Diseases Initiative Target Product Profile for Chagas Disease. https://dndi.org/diseases/chagas/target-product-profile/.

[50] Kratz J.M., Gonçalves K.R., Romera L.M.D., Moraes C.B., Bittencourt-Cunha P., Schenkman S., Chatelain E., Sosa-Estani S. (2021). The translational challenge in chagas disease drug development. Mem. Inst. Oswaldo Cruz.

[51] Katsuno K., Burrows J.N., Duncan K., Hooft van Huijsduijnen R., Kaneko T., Kita K., Mowbray C.E., Schmatz D., Warner P., Slingsby B.T. (2015). Hit and lead criteria in drug discovery for infectious diseases of the developing world. Nat. Rev. Drug Discov..

[52] El-sayed N.M., Myler P.J., Bartholomeu D.C., Nilsson D., Aggarwal G., Westenberger S.J., Tran A., Ghedin E., Worthey E.A., Delcher A.L. (2005). The Genome Sequence of Trypanosoma cruzi, Etiologic Agent of Chagas Disease. Science.

[53] Roy A., Kucukural A., Zhang Y. (2011). I-TASSER: A unified platform for automated protein structure and function prediction. Nat. Protoc..

[54] Biasini M., Bienert S., Waterhouse A., Arnold K., Studer G., Schmidt T., Kiefer F., Cassarino T.G., Bertoni M., Bordoli L. (2014). SWISS-MODEL: Modelling protein tertiary and quaternary structure using evolutionary information. Nucleic Acids Res..

[55] Pettersen E.F., Goddard T.D., Huang C.C., Couch G.S., Greenblatt D.M., Meng E.C., Ferrin T.E. (2004). UCSF Chimera--a visualization system for exploratory research and analysis. J. Comput. Chem..

[56] Irwin J.J., Shoichet B.K. (2005). ZINC—A free database of commercially available compounds for virtual screening. J. Chem. Inf. Model..

[57] Ochoa R., Watowich S.J., Flórez A., Mesa C.V., Robledo S.M., Muskus C. (2016). Drug search for leishmaniasis: A virtual screening approach by grid computing. J. Comput. Aided. Mol. Des..

[58] Huang B., Schroeder M. (2006). LIGSITE csc: Predicting ligand binding sites using the Connolly surface and degree of conservation. BMC Struct. Biol..

[59] Morris G.M., Huey R., Lindstrom W., Sanner M.F., Belew R.K., Goodsell D.S., Olson A.J. (2009). AutoDock4 and AutoDockTools4: Automated docking with selective receptor flexibility. J. Comput. Chem..

[60] Trott O., Olson A.J. (2009). Software News and Update AutoDock Vina: Improving the Speed and Accuracy of Docking with a New Scoring Function, Efficient Optimization, and Multithreading. Wiley Intersci..

[61] Daigneault M., Preston J.A., Marriott H.M., Whyte M.K.B., Dockrell D.H. (2010). The identification of markers of macrophage differentiation in PMA-stimulated THP-1 cells and monocyte-derived macrophages. PLoS ONE.

[62] Buckner F.S., Verlinde C.L.M.J., La Flamme A.C., Van Voorhis W.C. (1996). Efficient technique for screening drugs for activity against Trypanosoma cruzi using parasites expressing β-galactosidase. Antimicrob. Agents Chemother..

[63] Tolosa L., Donato M.T., Gómez-Lechón M.J., Vinken M., Rogiers V. (2015). General Cytotoxicity Assessment by Means of the MTT Assay. Protocols in In Vitro Hepatocyte Research.

[64] Tavares G.D.S.V., Mendonça D.V.C., Lage D.P., Granato J.D.T., Ottoni F.M., Ludolf F., Chávez-Fumagalli M.A., Duarte M.C., Tavares C.A.P., Alves R.J. (2018). Antileishmanial Activity, Cytotoxicity and Mechanism of Action of Clioquinol against Leishmania infantum and Leishmania amazonensis Species. Basic Clin. Pharmacol. Toxicol..

[65] Wu D., Yotnda P. (2011). Production and Detection of Reactive Oxygen Species (ROS) in Cancers. JoVE.

[66] D’Argenio D.Z., Schumitzky A., Wang X.A. (2009). ADAPT 5 User’s Guide: Pharmacokinetic/Pharmacodynamic Systems Analysis Software. https://bmsr.usc.edu/software/adapt/users-guide/.

[67] Akaike H. (1974). A new look at the statistical model identification. IEEE Trans. Automat. Contr..

