# Supplementary material for: In Silico, In Vitro, and Pharmacokinetic Studies of UBMC-4, a Potential Novel Compound for Treating against Trypanosoma cruzi
Source: Pathogens. 2022 May 24;11(6):616. doi: 10.3390/pathogens11060616 (PMC9229894; doi:10.3390/pathogens11060616)
Supplement: Supplementary file 1 [file pathogens-11-00616-s001.zip › pathogens-1719240-supplementary/Supplementary Material/supplementary.v3.pdf]

Supplementary Information

1. Absorption spectrum of UBMC-4

UBMC-4's absorption spectrum was evaluated over a wavelength range from 200 nm to 700 nm. Maximal absorption was observed in the region from 300 nm to 350 nm.

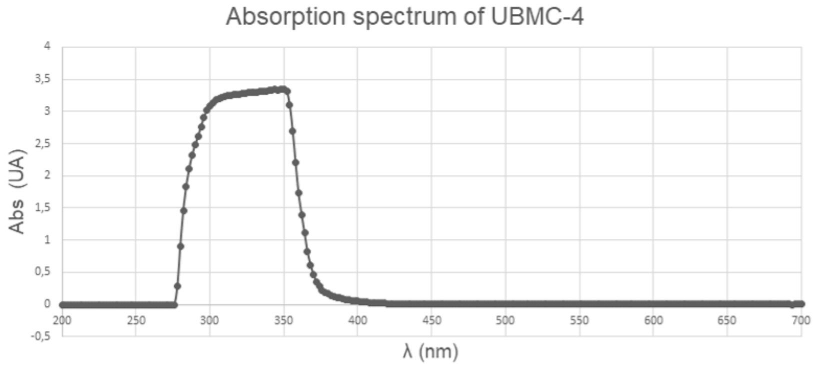

Figure S1. Absorption spectrum of UBMC-4.

2. HPLC method

A reverse-phase HPLC method was developed to quantify UBMC-4 in mice plasma.

Column: C18
λ: 300 nm
T: 40°C
Total time: 13 min
Mobile phase:

Table S1. Gradient HPLC method

Time (min)	A (%)	B (%)	C (%)	Flow (mL/min)
0	30	40	30	1.0
6	30	40	30	1.0
7	20	0	80	1.0
10	20	0	80	1.0
11	30	40	30	1.0

Where, A: Acetonitrile (0.1% TFA) – B: Water (0.1% TFA) – C: Methanol

2.1 Calibration curve

Table S2. Calibration curve of UBMC-4.

Compound	Level	Retention time	Quantity	Area	Height
UBMC-4	1	10,8233	20	2.074,2031	429,0315
		10,8267		2.105,1440	436,7761
		10,8333		2.052,8533	430,0585
	2	10,8233	10	1.157,3251	242,6668
		10,8317		1.153,5895	244,4369
		10,8283		1.172,1451	243,3595
	3	10,8150	5	1.025,7137	213,6284
		10,8267		1.039,2709	219,8506
		10,8283		1.046,6154	213,7983
	4	10,8150	2	239,3912	49,0692
		10,8283		249,8886	52,5522
		10,8183		242,8956	51,1904
	5	10,8250	1	84,5370	16,5988
		10,8283		130,2286	27,3586
	6	10,8267	0,5	28,3791	5,1623
		10,8233		51,5323	10,3720
		10,8200		62,9588	11,6813

Equation: Y=111.19946*X

$R^2 = 0.96985$

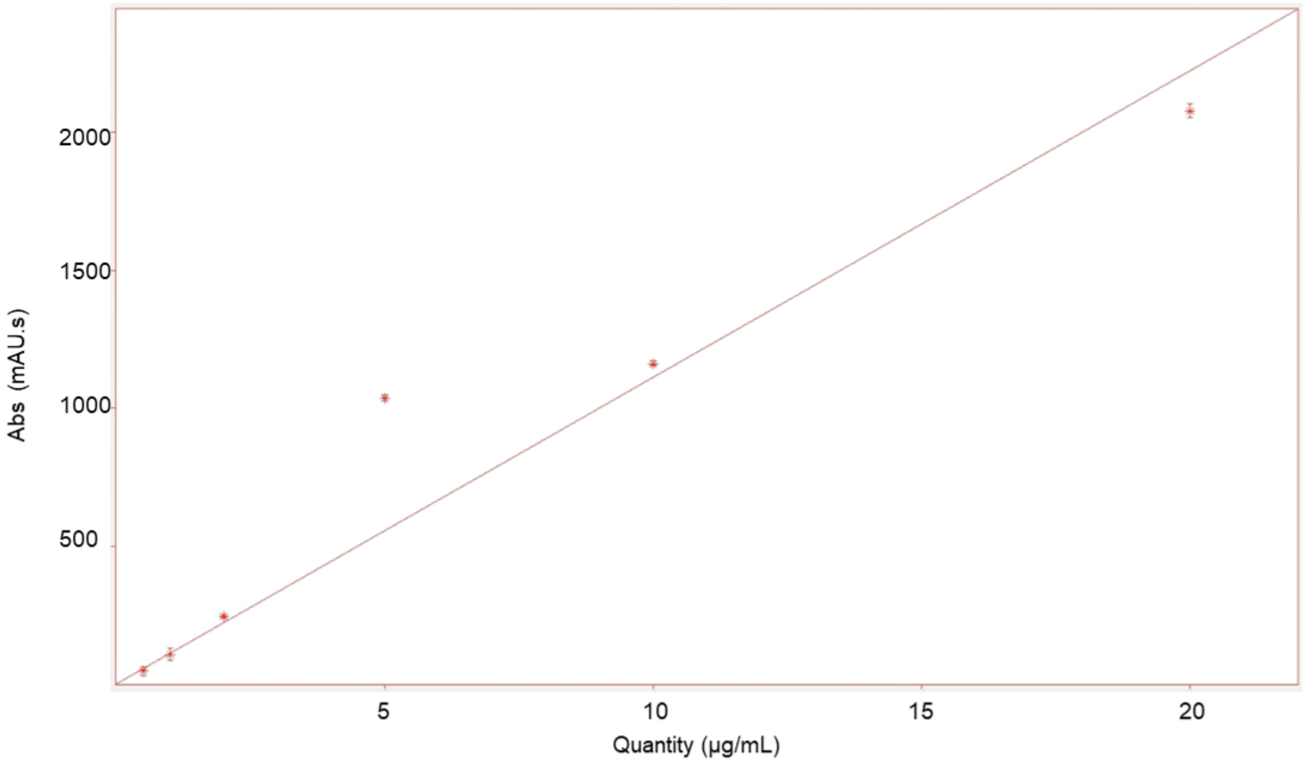

Figure S2. Calibration curve of UBMC-4.

2.2 Selectivity

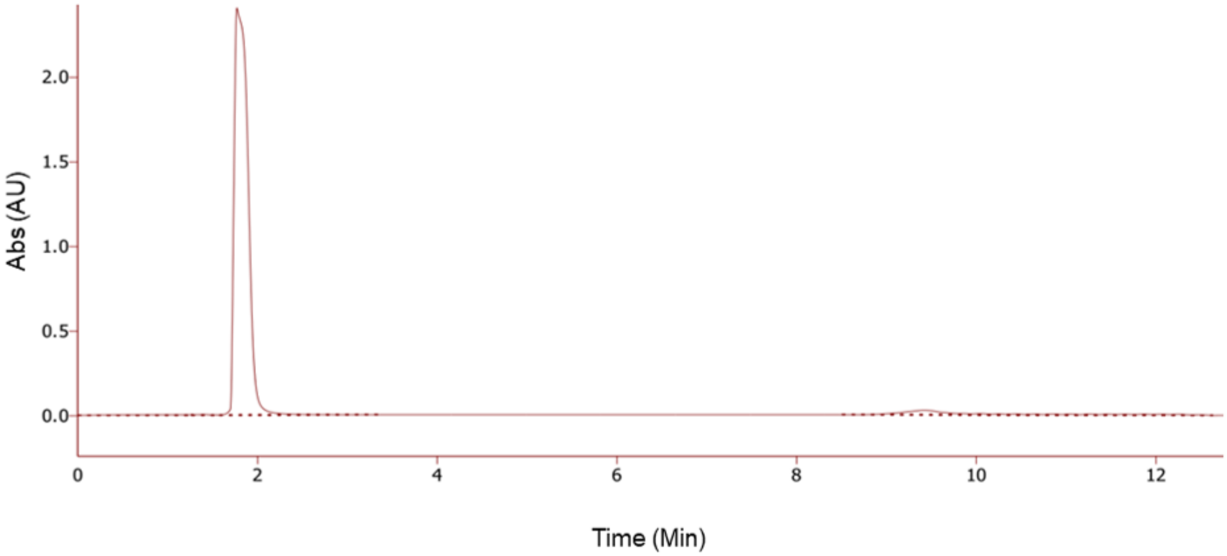

Figure S3-A. HPLC method selectivity. Mouse plasma + vehicle chromatogram. No peaks were observed.

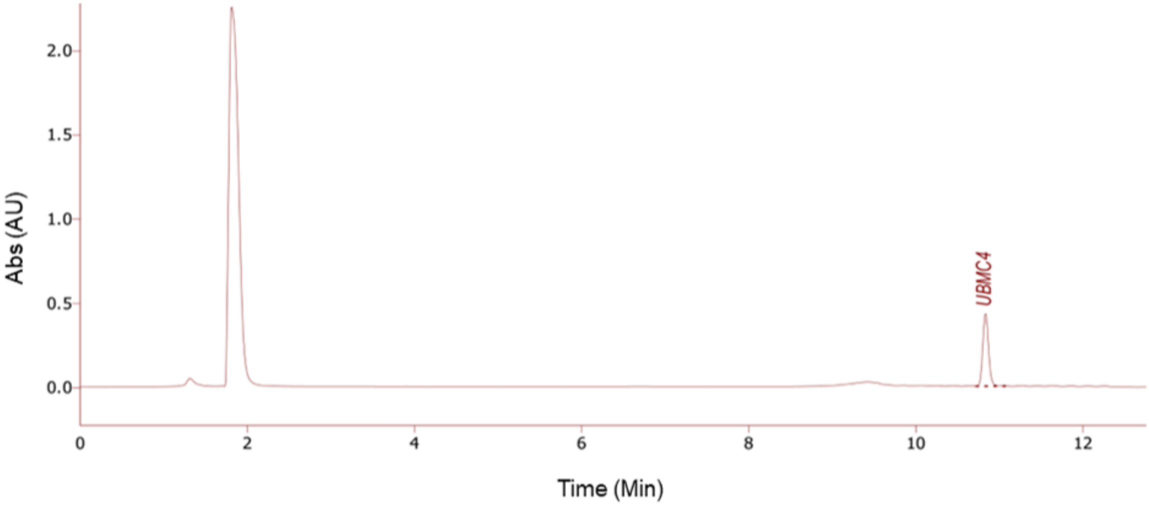

Figure S3-B HPLC method selectivity. Mouse plasma + vehicle + 0.1 mg/mL UBMC-4 chromatogram. Resolved peak corresponding to UBMC-4.
